# Integrated analysis of single-cell sequencing and machine learning identifies a signature based on monocyte/macrophage hub genes to analyze the intracranial aneurysm associated immune microenvironment

**DOI:** 10.3389/fimmu.2024.1397475

**Published:** 2024-06-24

**Authors:** Yifan Xu, Pin Guo, Guipeng Wang, Xiaojuan Sun, Chao Wang, Huanting Li, Zhenwen Cui, Pining Zhang, Yugong Feng

**Affiliations:** ^1^ Department of Neurosurgery, The Affiliated Hospital of Qingdao University, Qingdao, China; ^2^ Department of Urology, The Affiliated Hospital of Qingdao University, Qingdao, China; ^3^ Department of Oncology, The Affiliated Hospital of Qingdao University, Qingdao, China; ^4^ Department of Radiology, The Affiliated Hospital of Qingdao University, Qingdao, China

**Keywords:** intracranial aneurysm, single-cell sequencing, machine learning, immune microenvironment, hub genes

## Abstract

Monocytes are pivotal immune cells in eliciting specific immune responses and can exert a significant impact on the progression, prognosis, and immunotherapy of intracranial aneurysms (IAs). The objective of this study was to identify monocyte/macrophage (Mo/MΦ)-associated gene signatures to elucidate their correlation with the pathogenesis and immune microenvironment of IAs, thereby offering potential avenues for targeted therapy against IAs. Single-cell RNA-sequencing (scRNA-seq) data of IAs were acquired from the Gene Expression Synthesis (GEO) database. The significant infiltration of monocyte subsets in the parietal tissue of IAs was identified using single-cell RNA sequencing and high-dimensional weighted gene co-expression network analysis (hdWGCNA). The integration of six machine learning algorithms identified four crucial genes linked to these Mo/MΦ. Subsequently, we developed a multilayer perceptron (MLP) neural model for the diagnosis of IAs (independent external test AUC=1.0, sensitivity =100%, specificity =100%). Furthermore, we employed the CIBERSORT method and MCP counter to establish the correlation between monocyte characteristics and immune cell infiltration as well as patient heterogeneity. Our findings offer valuable insights into the molecular characterization of monocyte infiltration in IAs, which plays a pivotal role in shaping the immune microenvironment of IAs. Recognizing this characterization is crucial for comprehending the limitations associated with targeted therapies for IAs. Ultimately, the results were verified by real-time fluorescence quantitative PCR and Immunohistochemistry.

## Introduction

IAs are focal deformities commonly found in branches of cerebral arteries, with a prevalence of 2% - 5% of the total population (1). IAs are usually in a stable state but may rupture, with a lesion incidence of 1.2% per patient per year (2). Subarachnoid hemorrhage caused by ruptured intracranial aneurysms leads to serious complications and even life-threatening conditions (3, 4). About 30% of IAs patients die from Subarachnoid hemorrhage (5). Endovascular therapy and surgical clamping are currently the primary means of treating aneurysms, but they are expensive and carry a significant risk of complications (6), and the use of medication may present a viable alternative for individuals with a heightened susceptibility to undergoing invasive surgical procedures (7, 8). However, therapeutic drug targets are currently lacking. Therefore, there is a pressing need to find new biomarkers that could help to explore the molecular mechanism of the formation and progression of IAs, which will aid in developing new treatment strategies.

The main histopathological characteristics of IAs include immune infiltration, cell death, oxidative stress, lipid metabolism, iron accumulation and proteolytic activity (9–11). The pathogenesis of IAs has been extensively investigated, and numerous studies have consistently demonstrated the involvement of various immune cell types. The effects of macrophage-driven inflammation on the aneurysm wall were summarized by Sajjad et al. (12), who also discussed pharmacological strategies for modulating the macrophage response during Internal carotid aneurysm formation and rupture. Hajime et al. (13)induced intracranial aneurysms in adult mice and subsequently administered a mast cell stabilizer (sodium glycyrrhizinate) along with a mast cell activator (C48/80). Their findings demonstrated that the pharmacological stabilization of mast cells through sodium glycyrrhizinate significantly reduced the incidence of aneurysm rupture. The increasingly significant role of immune infiltration in aneurysms presents novel opportunities for comprehending the development of IAs. In addition, CD4 T cells (14) and lymphocytes (15) were also demonstrated. The research on Mo/MΦ remains limited, with few studies conducted thus far. Mo/MΦ plays a crucial role in the body’s non-specific immune defense by presenting antigens, initiating immune responses, and secreting a diverse array of cytokines to actively participate in immune regulation. The key cytokines involved include IL-1 and IL-12 (16). The field of Mo/MΦ-based therapies has witnessed significant advancements over the decades, establishing it as a pivotal avenue for future research.

While numerous studies have conducted comprehensive experiments to elucidate the molecular mechanisms underlying the formation and progression of IAs, only a limited number of studies have utilized scRNA-seq to identify potential targets that may play crucial biological roles in these mechanisms. RNA-seq employs optimized next-generation sequencing techniques to define the overall gene expression profile of individual cells, thereby facilitating the dissection of heterogeneity within previously concealed cell populations. In this study, we integrated scRNA-seq data with batch RNA-seq data from IAs to discern differences between distinct cell clusters. We employed hdWGCNA and machine learning approaches to characterize the immune landscape and screen for immune-related hub genes implicated in the development of IAs. Finally, we combined 4 key genes and 10 immune cell types to synthesize personalized portraits and establish a gene immunoconvolutional neural network deep learning model for precise diagnosis and treatment of IAs.

## Methods

### Data collection

The scRNA-seq date in GSE193533 and bulk-seq data in GSE75436 and GSE122897 were downloaded from the GEO database (https://www.ncbi.nlm.nih.gov/geo/), A total of 61 IAs samples and 21 control samples were included, GSE193533 contains two IAs samples and one control sample, and GSE75436 contained 15 IAs samples and 15 superficial temporal artery samples. GSE75436 contains 44 IAs samples and 16 control samples. Moreover, the GSE75436 dataset was designated as the discovery dataset, and GSE122897 were utilized as the validation datasets. The patient’s information is shown in **Supplementary Tables S1**, **S2**.

### scRNA-seq data processing

The scRNA-seq data was initially processed using the Seurat package to preserve high-quality scRNA-seq data. Subsequently, The original matrix of each cell was subjected to three filtering measures: retaining a minimum of five genes expressed by single cells, excluding cells expressing fewer than 100 genes, and excluding any cells expressing more than 20% of mitochondrial genes. Next, the initial set of 2,000 highly variable genes was identified using the “Discover Variant features” function within the “Seurat” package. Subsequently, principal component analysis (PCA) was conducted on these 2000 highly variable genes using the “RunPCA” function to reduce dimensionality in scRNA-Seq data. Cell clustering was performed utilizing the “Find Neighbor” and “Find Cluster” functions available in the Seurat package (17).

### The pipeline of high-dimensional WGCNA

The hdWGCNA method was employed to construct a cell-type-specific co-expression network, followed by the identification of gene modules and co-expressed genes within the network (18). Hierarchical clustering and dynamic cutting tree function were utilized for module identification, with different branches representing distinct gene modules. Hub genes were selected based on their gene significance (GS) and membership degree (MM) in the module. The analysis steps are as follows: firstly, the gene expression data were preprocessed. Secondly, the gene co-expression network was constructed. Thirdly, modules or clusters of highly correlated genes are identified, module signature genes are calculated, and module preservation analyses are performed to assess the robustness of the identified modules. Finally, in the fifth step, the functional enrichment analysis is carried out.

### Pseudotime analysis and cell communication analysis

The Monocle2 algorithm was used to reveal the change rule of key gene expression over time, and even the hidden change pattern was found. The size factor and dispersion are first estimated, and highly variable features are determined within the Monocle object. The cell differentiation state was determined by the DDRtree method, and then the cell differentiation trajectory was visualized. To gain a comprehensive understanding of the specific cellular interactions, we utilized the CellChat package to infer and analyze cell-cell communication (19, 20). The analysis of cell-cell interactions was conducted separately for IAs and normal groups, with a focus on evaluating the major signal inputs and outputs using CellChatDB (21).

### Functional enrichment analysis

The highly correlated genes identified by hdWGCNA were subjected to enrichment analysis using gene Ontology (GO), Disease Ontology (DO) and Kyoto Encyclopedia of Genes and Genomes (KEGG). Functional enrichment was assessed utilizing the “GOplot”, “Cluster Analyzer”, and “DOSE” packages in R.

### Friends analysis and protein-protein interaction analysis

The construction of an immune gene-associated PPI network was facilitated using the STRING database (https://string-db.org/). The Friends analysis method was employed to examine genes exhibiting strong correlations with other genes within the same pathway, and R-packet GOSemSim (21) was used to calculate functional correlation scores between key genes.

### Identification of potential key genes by machine learning algorithms

The feature selection of disease diagnosis factors in this study employed the machine learning algorithms random forests (RF), support vector machines (SVM), least absolute shrinkage and selection operator (LASSO), neural network (NNET), Boruta and k‐nearest neighbor (KNN). LASSO regression is characterized by fitting a generalized linear model and screening variables, which analysis was realized by glmnet software package with 10-fold cross-verification through a turning/penalty parameter (22). SVM-RFE is a machine learning algorithm based on the maximum interval theorem of SVM. It adopts the principle of minimizing structural risks and minimizing empirical errors, to strengthen the learning performance (23). The SVM module was developed by the “e1071” package. RandomForest is used to rank genes. The Boruta algorithm is a supervised classification feature selection method utilized for the identification of all relevant features in a classification task. The NNET model implements a nonlinear mapping function that effectively captures the relationship between input and output data through learning, while also adaptively storing this knowledge in its network weights. It exhibits robust generalization capabilities and fault tolerance. On the other hand, KNN is a non-parametric delayed learning architecture that leverages Euclidean distance to classify instances based on their proximity to K neighboring data points (23). Ultimately, we combine six machine learning modes to further screen the most significant feature genes. The expression levels of these candidates were subsequently validated to assess their potential as diagnostic biomarkers.

### The functionally-related genes

The initial selection of the top 20 genes interacting with the four key genes was performed using machine learning techniques on the GeneMANIA database (http://genemania.org/) with default parameters.

### MLP neural network architecture

The basic structure of MLP comprises three layers: the input layer, the hidden layer, and the output layer. MLP neural networks establish full connectivity between different layers, and the hidden layer is divided into three layers. For the input layer and hidden layer, The ReLU activation function is utilized for the model, while the output layer utilizes the softmax activation function. The Adam optimizer is employed with a learning rate of 0.0001., and the classification cross-entropy loss function is used to evaluate performance. Model accuracy is determined through analysis of predicted versus actual class labels using a confusion matrix. Keras was utilized for machine learning analysis (24).

### The assessment and analysis of infiltration by immune cells

The CIBERSORT is a commonly used method for deconvoluting immune cell expression matrices based on linear support vector regression, enabling the quantification of infiltrated immune cells through gene expression labeling (25). In our study, we utilized marker gene expression and transcriptome data from 28 immune cell types to accurately determine the distribution values of monocytes through CIBERSORT analysis. The infiltration level of immune cells and immune-related pathways were assessed using single sample gene set enrichment analysis (ssGSEA), based on the expression profiles of 28 immune-related signals. Heat maps depicting the distribution and changes in immune cell populations were generated using the “ggplot2” software package. Additionally, Microenvironment Cell Population Counting (MCP-cecter), a genomic signature-based approach, was employed to estimate the abundance of infiltrating immune and stromal cells from transcriptome data (26).

### Gene set enrichment analysis

The GSEA analysis determines whether all genes in a gene set exhibit clustering at the top or bottom of an ordered list, indicating up-regulation or down-regulation of the gene set, respectively. The expression matrix of IAs and the control group was subjected to clustering using the R package GSEA. A significance level of FDR < 0.05 with 1000 random combination settings is considered statistically significant.

### Analysis of transcriptional factor regulatory network

The Single-cell Regulatory Network Interference and Clustering (SCENIC) tool enables the simultaneous reconstruction of gene regulatory networks and the identification of stable cell states from scRNA-seq data. The “GENIE3” package was used to infer gene co-expression networks, the “RcisTarget” package was used to analyze transcription factor binding motif, and the “AUCell” package was used to identify cells with active gene sets (gene networks) in the scRNA-seq data. This revealed the possible relationship between the four genes screened by machine learning and IAs.

### Patients and samples

A total of 10 patients diagnosed with IAs were recruited for this study, and complete vascular wall tissue samples were collected from the IAs patients who underwent microsurgical clamping. Ten segments of the superficial temporal artery were obtained during surgery for traumatic intracranial hemorrhage (controls matched in terms of sex, age, and blood pressure), detailed methods and matching data are provided in the **Supplementary Material 1**. The basic characteristics associated with immune conditions are shown in **Supplementary Table S3**. The tissue samples were rapidly frozen in liquid nitrogen and promptly transported to the laboratory for subsequent experiments. The study protocol obtained written informed consent from each patient before the commencement of the study and received approval from the Ethics Committee of the Affiliated Hospital of Qingdao University, The series number of approval documents is QYFY WZLL 28225.

### Quantitative real-time PCR

The extraction of total RNA from tissue samples was performed using Trizol reagents (Carlsbad, CA). The concentration of RNA was determined by ultraviolet spectrophotometry. Reverse transcription was carried out using the superscript RT-PCR First Chain Synthesis System Kit (Invitrogen) and random hmers. Real-time PCR of cDNA was performed using the Mx-3000P quantitative PCR system (Stratagene). The primer sequence for PCR is as follows: LGMN, CCATGCCTACCAGATCATTCAC (forward), GGTAACATCCTCTCCAGTGTAGTC (reverse); FN1, ACCTGGAGCAAGAAGGATAATCG (forward), GCATCCCCACAGAGTAGACC (reverse); SRGN, CTTCCCACTTTCTGAGGACTAC (forward), CTAACTACATTGCCTGGTGTCA (reverse); CXCL16, AGCGTCACTGGAAGTTGTTATTG (forward) AGCTGGAACCTCGTGTAGTATAG (reverse). The data visualization was performed using the GraphPad Prism 8 software.

### Immunohistochemistry

The expression of key genes was determined through IHC staining. Specifically, the sections were subjected to heat-induced antigen retrieval by heating, dewaxing, and rehydration. Subsequently, they were incubated overnight with antibodies against LGMN, FN1, SRGN, and CXCL16 in a 4°C humidifier box. Afterward, the slices were incubated at 25°C for 20 minutes with rabbit anti-goat antibody labeled with horseradish peroxidase. Following this step, the sections were stained using hematoxylin (Gene Tech, Shanghai China). Finally, visualization was performed using a Leica DM 2500 microscope. The specimens were subjected to examination under a light microscope.

### Statistical analysis

The unpaired T-test and Wilcoxon rank sum test were employed to analyze the differences in the data. The correlation of tissue expression levels was assessed using the Pearson correlation test. The statistical P-values were calculated using a two-tailed test, and p < 0.05 was deemed to be statistically significant. All analysis was conducted using R software version 4.1.3.

## Results

### Identification of IAs cell clusters

**Figure 1:**
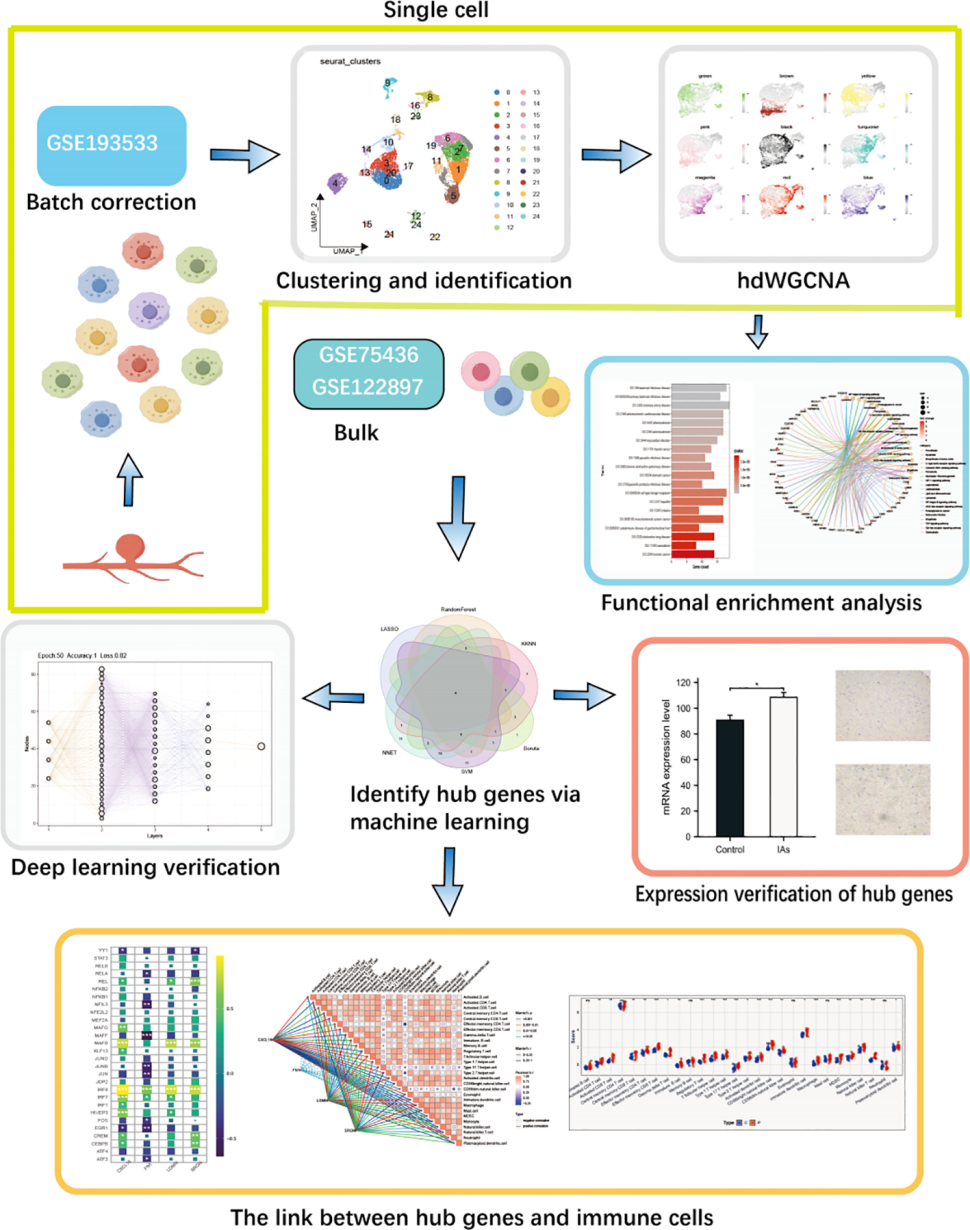
The complete workflow of this study.

**Figure 2:**
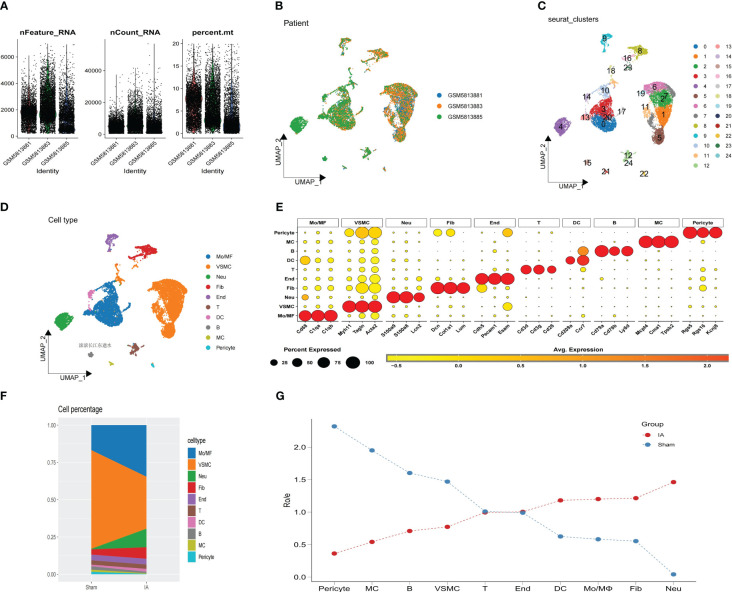
Analysis of single-cell RNA sequencing. **(A)** The sequencing depth from 3 IAs samples. **(B)** UMAP plot of 14,809 cells from 3 primary IAs samples. **(C)** UMAP plot colored by various cell clusters. **(D)** The cell types are identified by marker genes. **(E)** Dot plot showing representative marker genes for each cell type. **(F)** The proportion of each cell type from IAs and sham is shown, as indicated. **(G)** The line plots show the composition and changes of each cell in IAs and sham.

The process is depicted in the flowchart presented in **Figure 1**. The expression profile was used for this analysis, which contained 3 samples (GSM 5813881, GSM 5813883 and GSM 5813885), after filtering the unqualified cells, 14,809 core cells were obtained for subsequent analysis (**Figures 2A, B**). The UMAP algorithm was used to cluster their cells to obtain 24 clusters (**Figure 2C**), which were later translated to known cell types (**Figure 2D**). Subsequently, to transform to known cell types, annotated using standard cell markers (**Figures 2E, F**). The line plots show the composition and changes of each cell in IAs and sham (**Figure 2G**). The visualization of key marker genes for each cell type was achieved through the utilization of bubble plots (**Supplementary Figure 1A**).

### Heterogeneity between cell clusters of IAs

**Figure 3:**
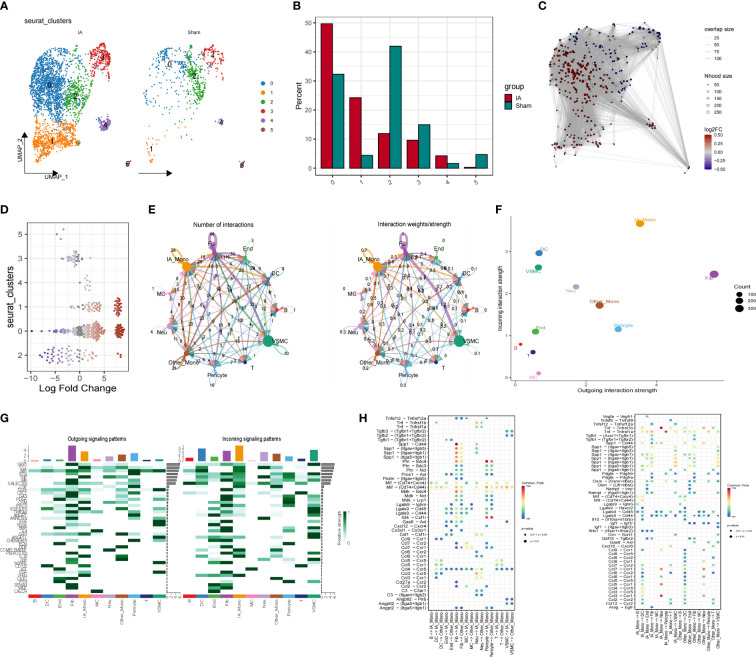
Altered proportions of Mo/MΦ subpopulations in IAs. **(A)** The UMAP scatter plot illustrates the spatial distribution of the Mo/MΦ subtype. **(B)** The stacked bar chart depicts the proportion of Mo/MΦ subtypes. **(C)** UMAP for scWB scRNA-seq differential abundance in samples from patients with IAs compared to HCs, with sampled neighborhoods colored by statistical significance (spatial FDR < 0.05). Nhood, neighborhood. **(D)** Beeswarm plots of differential cell abundance in scWB with cluster labels of neighborhoods depicted and compared for IAs and normal samples. **(E)** Strength and number of interactions between key cells. **(F)** The IAs cell communication network is visualized in terms of signal communication. **(G)** Interaction quantity and interaction weight/strength of various cells in the IAs in the communication network. **(H)** Bubble plots show specific pathways by which different cell types exhibit stronger interactions.

We compared the difference in the proportion of cell types in the IAs and sham groups using UMAP diagrams. We further dissected the monocyte subpopulation and showed that not all monocytes were universally upregulated, with only cluster 0 demonstrating a significant increase (**Figures 3A, B**). We performed fine-resolution clustering and annotation and applied the miloR tool to quantify shifts in the abundance of all cell types between groups (**Figures 3C, D**). The cell-cell communication analysis was performed using the cellchat pipeline. In detail, the frequency and intensity of interactions between IA_Monocyte and Pericyte, between VSMC and fibroblasts, and between other_Monocyte and DC cells were high (**Figure 3E**). **Figure 3F** shows that IA_Mono and Other_Mono exhibit enhanced output and input interaction strength. Moreover, IA_Monocyte sent and received more signals to other cells mediated by INF-1, while Other_Monocyte sent more signals to other cells mediated by IGE and received more signals mediated by CX3C (**Figure 3G**). We then analyzed and compared the specific pathways by which various cell types exhibit stronger interactions (**Figure 3H**), the results showed that fibroblasts sent stronger signals to IA_Mono in the Spp1-related pathway compared to Other_Mono, and IA_Mono sent stronger signals to IA_Mono in the CCL-related pathway compared to Other_Mono.

### HdWGCNA identifies hub genes associated with Mo/MΦ

**Figure 4:**
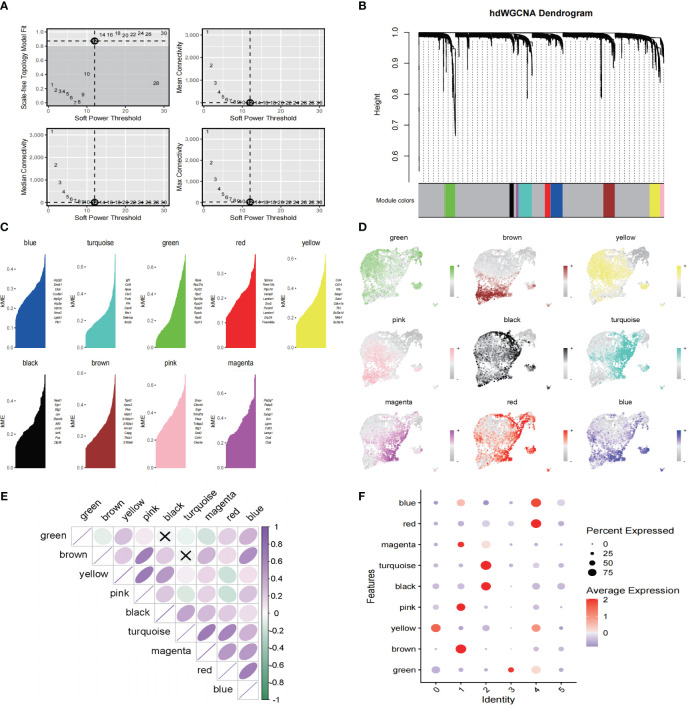
hdWGCNA has revealed the key Mo/MΦ genes in the pathogenesis of IAs **(A)** Select a soft power supply suitable for running the hdWGCNA, the mean value, median value and maximum connectivity of the topology network are shown respectively when different minimum soft thresholds are selected. **(B)** Nine modules are identified as shown in the tree diagram. **(C)** Each module presents the top hub gene. **(D)** The feather plot depicts the score for nine modules. **(E)** Correlation analysis between different models. **(F)** Bubble plot reveals the scores obtained by each module.

To investigate the intrinsic functions and properties of Mo/MΦ, we constructed a co-expression network based on single-cell data using an optimal soft threshold of 12, as illustrated in **Figure 4A**. Nine gene modules were generated (**Figure 4B**). **Figure 4C** presents the top 10 hub genes for these nine modules. Genes highlighted in yellow and brown within the gene modules exhibited a higher likelihood of expression in clusters 0 and 1, respectively, demonstrating a significant positive correlation (**Figure 4D**). In addition, the blue module shows A strong positive correlation in the red module, the pink module shows a strong positive correlation with the yellow and brown module, and the turquoise module has a strong positive correlation with the magenta module (**Figure 4E**). We then scored the genes of the top 25 hub genes ranked by kME in each module (**Figure 4F**).

### Pseudo-time and trajectory analysis

**Figure 5:**
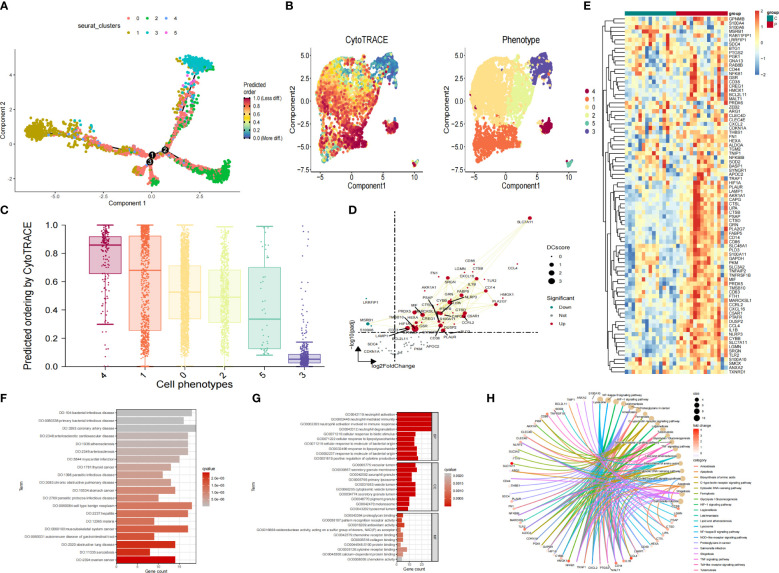
Pseudotime analysis, trajectory analysis and analyses of functional enrichment of DEGs. **(A)** pseudotime distribution of the different macrophage subtypes **(B)** Pseudotime analysis based on the monocle3 package shows the differentiation trajectory of IAs cell subtypes. **(C)** The box diagram reflects the relationship between cpredicted ordering by cyto trace and cell phenotype. **(D)** Scatter plot showcasing differential analysis results between high-risk cells and background cells within Mo/MΦ. **(E)** Heat maps showed the expression of genes in IAs group and sham group. **(F)** DO analysis of co-expressed genes. **(G)** GO analysis of co-expressed genes. **(H)** KEGG analysis of co-expressed genes.

To determine the transcriptional characteristics of Mo/MΦ at different stages of development, we performed a pseudo-temporal analysis. Cells that have similar states are grouped, and branch points divide cells into different states. Notably, cluster 0 Mo/MΦ are mainly located at the end of the pseudo-time locus (**Figure 5A**). We further applied CytoTRACE analysis predict the origin of Mo/MΦ by integrating Monocle2 (**Figure 5B**). Then the relationship between cpredicted ordering by cyto trace and cell phenotype was analyzed (**Figure 5C**). Gene expression analysis of IAs sample in cluster 0 compared with control sample showed that SLC7A11, CXCL16, SRGN, FABP5, CD14 and CYBB genes were significantly up-regulated in IAs (**Figure 5D**). Finally, Heat maps showed that most genes were up-regulated in the IAs group and down-regulated in the control group (**Figure 5E**).

### The genes in the yellow and brown modules was analyzed for functional enrichment

The function of genes in the yellow and brown modules was investigated by conducting an enrichment analysis on the top 50 hub genes within these modules, resulting in a total of 100 genes (**Supplementary Table S4**) in the yellow and brown modules. The consequences of DO analysis illustrate that these common genes are relevant to, coronary artery disease, cell type benign neoplasm and bacterial infectious disease (**Figure 5F**). The GO analysis revealed that hub genes were significantly enriched in processes related to neutrophil activation, neutrophil-mediated immunity, and immune response involving neutrophil activation (**Figure 5G**). Additionally, functional enrichment analysis was conducted for the candidate genes, and KEGG analysis displayed that “Proteoglycans in cancer”, “Lysosome”, “NF−kappa B signaling pathway” and “HIF−1 signaling pathway” pathways depicted predominant enrichment of genes (**Figure 5H**).

### Visualize hub gene expression levels

**Figure 6:**
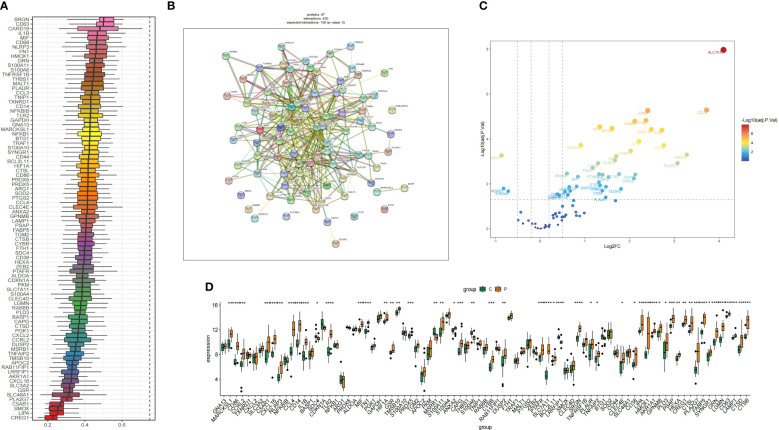
The correlation analysis of yellow and brown module genes was carried out. **(A)** Friends analysis of genes in the yellow and brown modules, the total score ranges from 0 to 1, with higher scores indicating more genes associated with it. **(B)** PPI of genes in the yellow and brown modules. **(C)** The volcano map shows the expression analysis of potential characteristic genes between IAs and sham samples. **(D)** The bar chart shows the levels of gene expression in the IAs group and the shame group. *P < 0.05, **P < 0.01, ***P < 0.001, ****P < 0.0001.

**Figure 6A** shows the results of the friends analysis of these 100 hub genes. The results showed that the eight genes with the strongest correlation were SRGN, CD63, CARD19, IL1B, MIF. We performed PPI analysis of 100 genes through the STRING database and visualized and mapped the results of protein interactions, 23 genes were found to have strong biological functional similarities (**Figure 6B**). Next, we drew bar charts and volcano plot showing how much of the hub genes was expressed (**Figures 6C, D**).

### Identification and validation of hub genes by machine learning

**Figure 7:**
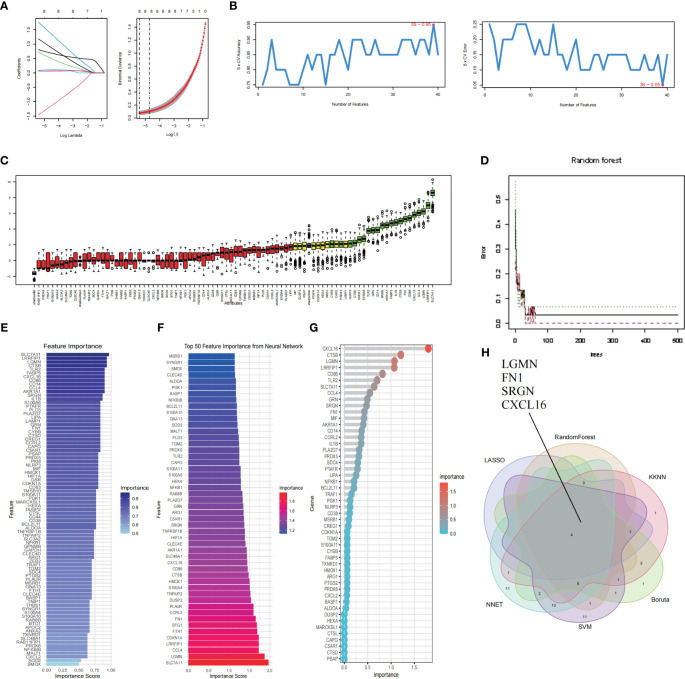
Identification and validation of hub genes by Machine learning. **(A)** LASSO regression shrunk the genes to 8. **(B)** The selection and validation of biomarker signature genes were performed using the SVM-RFE algorithm. **(C)** Boruta screened 21 feature genes for importance sequencing. **(D)** The correlation between the error rate of a random forest and the quantity of classification trees. **(E)** KNN algorithm was used for feature gene selection. **(F)** NNET algorithm was used for feature gene selection. **(G)** The top 50 relatively important genes in random forest. **(H)** The Venn diagram illustrates the common genes shared by LASSO, RF, SVM, NNET Boruta and KNN algorithms.

We select the 6 most important features and further screen the key genes with the most diagnostic value according to the machine learning algorithm. The LASSO analysis identified a total of 8 genes. (**Figure 7A**). We then applied five machine learning algorithms, RF, SVM-RFE, NNET Boruta and KNN (**Figures 7B–F**), and by overlapping them we ended up with 4 shared hub genes: LGMN, FN1, SRGN and CXCL16 (**Figure 7H**). **Figure 7G** shows the top 50 relatively important genes in RF.

### Assessment of the expression and diagnosis significance of hub genes

**Figure 8:**
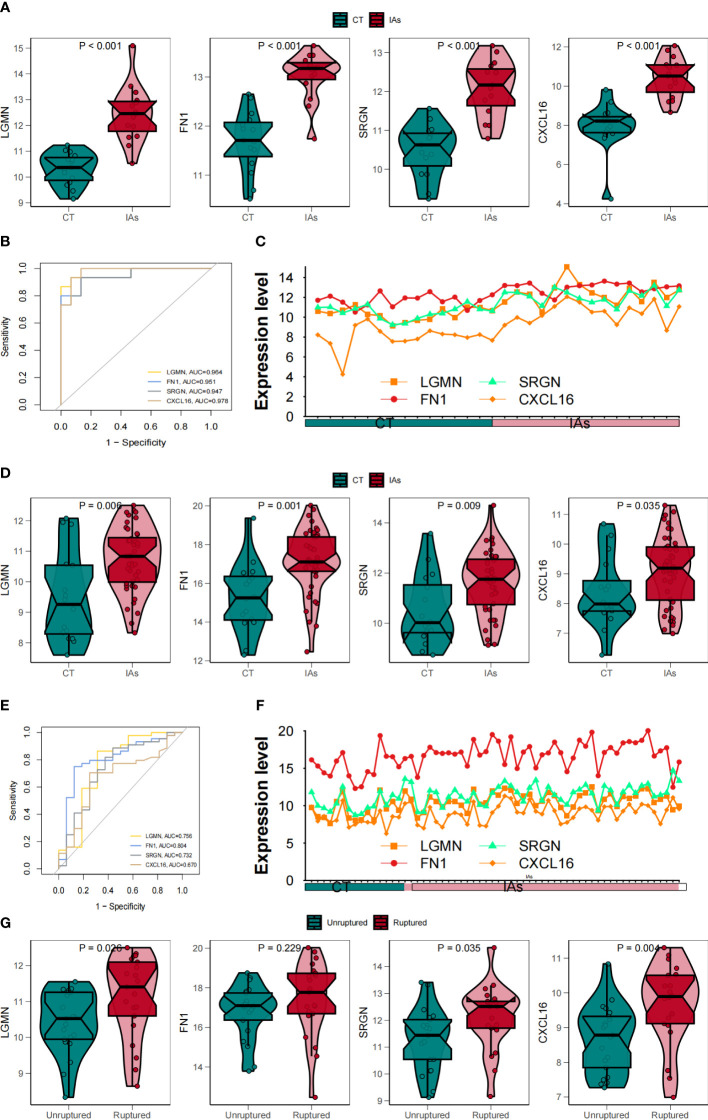
To verify the correlation between four core genes and Mo/MΦ. **(A)** Box line plots compared gene expression levels in the training set. **(B)** ROC curves of the feature genes in the training set. **(C)** The line plots compared gene expression levels in the training sets in the IAs and Shame groups. **(D)** Box line plots compared gene expression levels in validation sets. **(E)** ROC curves of the feature genes in the validation set. **(F)** The line plots compared gene expression levels in the validated sets in the IAs and Shame groups. **(G)** Differences in key fundamental expression between unruptured and ruptured IAs samples.

To further verify the diagnostic and prognostic efficacy of each shared central gene, we used ROC curve and expression curve for evaluation. To confirm the previous findings, we validated the expression differences of these four genes between samples of different states in two downloaded datasets, and GSE 75436 was used as the training set and GSE 122897 was used as the verification set. We observed that in the training set, LGMN, FN1, SRGN, and CXCL16 were significantly upregulated in the IAs group (**Figures 8A–C**). The same results are obtained for the verification set (**Figures 8D–F**). Subsequently, we found that the samples used included both ruptured and non-ruptured IAs samples, so we analyzed the differences between unruptured and ruptured aneurysms, and the results showed that ruptured IAs samples had higher levels of hub gene expression (**Figure 8G**).

### Construction of the gene-gene interaction network and correlation analysis of hub genes

The gene-gene interaction network of central genes was initially constructed, and the functional analysis of these genes was performed using the GeneMANIA database. Surrounding the central nodes were 20 additional nodes representing significant correlations with the central genes (**Supplementary Figure 2A**). Subsequently, an in-depth examination of the interrelationships among the four hub genes was conducted (**Supplementary Figure 2B**).

### Construction of the pre-biased multi-layer perceptron neural network model

**Figure 9:**
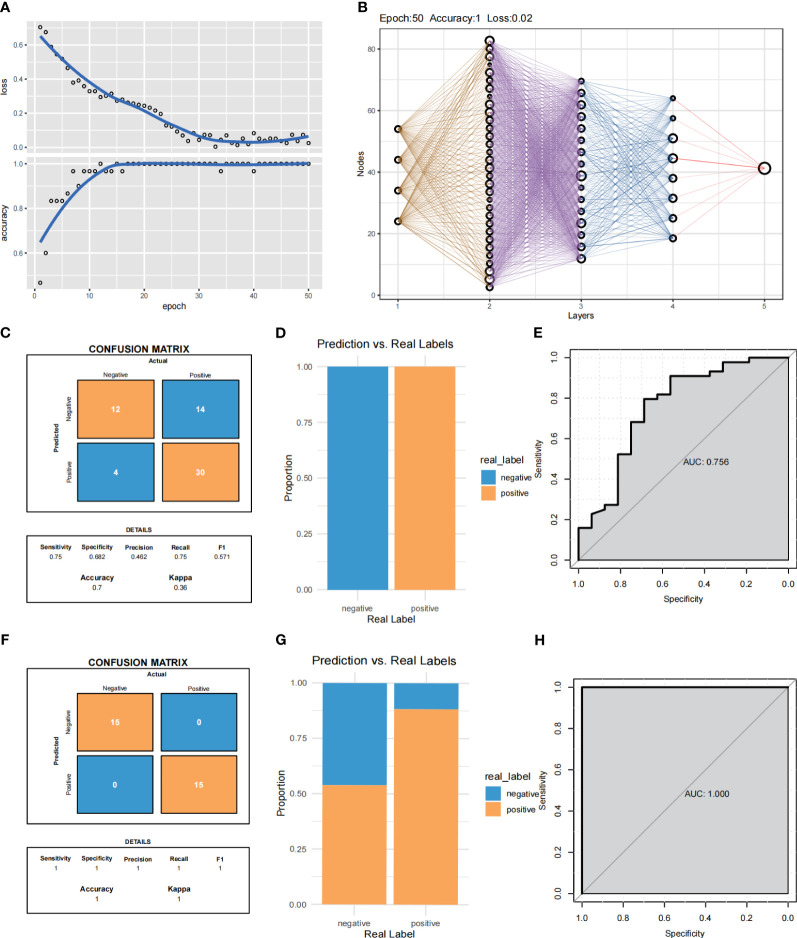
MLP neural networks predict Cox regression of feature selection used in e novo clusters. **(A)** Training process of the gene-immune MLP model. **(B)** Validation of the MLP neural network of the training group and validation group. **(C, D)** Summary of the samples utilized for validating the performance of the MLP neural network model within the training group. **(F, G)** Summary of the samples utilized for validating the performance of the MLP neural network model within the validation group. **(E, H)** ROC curves of the training group and validation group.

The convolutional neural networks were trained for 200 epochs (**Figure 9A**). By integrating these selected feature inputs with the initial weights, the feedforward structure of three hidden layers facilitates easy classification of one of the four clusters in the output layer (**Figure 9B**). The expression of these four genes was significantly different in the IAs sample and the shame sample, and the IAs sample and the shame sample could be distinguished by these four genes, with an accuracy of 70% in the training group and 100% in the validation group (**Figures 9C, D, F, G**). The MLP neural network is constructed with 1467 features, which are selected by the univariate Cox regression model and used as input to the nodes. The model demonstrates excellent predictive accuracy in both datasets (**Figures 9E, H**, training AUC = 0.756, testing AUC = 1.000).

### Signaling pathways involved in characteristic genes

The GSEA method was employed to conduct an analysis of the specific signaling pathways associated with four hub genes. The pathways associated with the central gene of utmost significance were selected, as depicted in **Supplementary Figure 2**. The results demonstrated a significant upregulation and enrichment of CXCL16 in Viral protein interaction with cytokine and cytokine receptor, Systemic lupus erythematosus, Renin−angiotensin system and Pertussis (**Supplementary Figure 3A**). High expression of FN1 enriched in Nicotine addiction, Cocaine addiction, Insulin secretion and Aldosterone−regulated sodium reabsorption (**Supplementary Figure 3B**). Systemic lupus erythematosus, Asthma, Allograft rejection and Intestinal immune network for IgA production were mainly enriched in LGMN (**Supplementary Figure 3C**). Pantothenate and CoA biosynthesis, Pertussis, Viral protein interaction with cytokine and cytokine receptors, Graft−versus−host disease and Legionellosis were mainly enriched in SRGN (**Supplementary Figure 3D**).

### Correlation analysis between hub genes and immune characteristics

**Figure 10:**
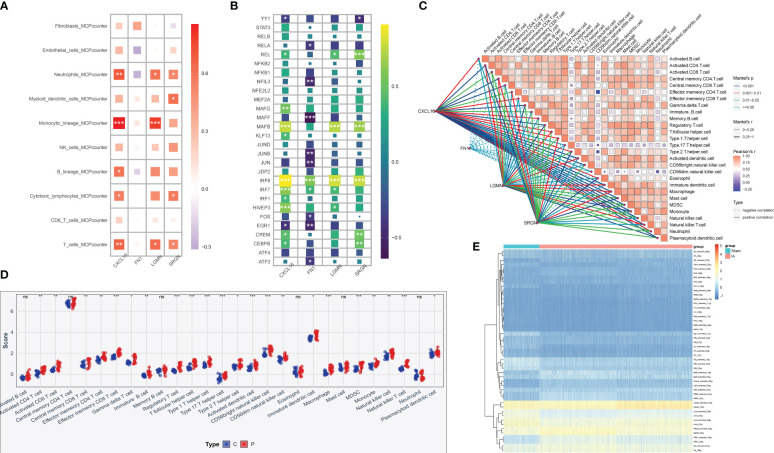
Immune infiltration correlation analysis of hub genes. **(A)** By using MCP-counter analysis, the heat map shows the correlation between the expression levels of four genes screened by machine learning and the type of immune cells. **(B)** Correlation between hub gene expression and 28 immune pathways. *p < 0.05, **p < 0.01, ***p < 0.001, ns: no significance. **(C)** Correlation analysis of risk scores with significantly different immune cells. **(D)** The abundance of 28 immune-related cells differs in IAs tissue and normal tissue. **(E)** Heat map compared the associations with immune pathways in the experimental and control groups.

The association of four central genes with immune cells was analyzed by MCP-counter and the results were visualized, the results were consistent with our expectation that monocytes were highly correlated with CXCL16 and LGMN (**Figure 10A**). Furthermore, immune characteristics were evaluated according to immune checkpoint and immune cell infiltration expression (**Figure 10B**), and the results illustrated that IRF8 signal transduction pathway was highly correlated with the four keys, and MAF signal transduction pathway was highly correlated with CXCL16, LGMN, and SRGN. In addition, we calculated correlations between the IAs samples and immune cells, and most of the sites examined were significantly upregulated in patients with IAs compared to the normal population (**Figures 10C, D**). Finally, the relationship between the experimental group and the control group and the immune pathway was compared by heat maps (**Figure 10E**).

### Validated the expression of the four model-related genes

**Figure 11:**
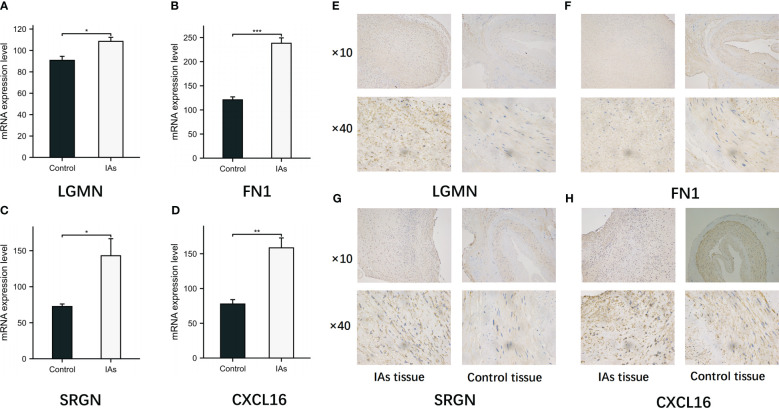
Expression of LGMN, FN1, SRGN and CXCL16 in IAs tissues and control tissues. **(A-D)** The mRNA levels of LGMN, FN1, SRGN and CXCL16 in control group and observation group were detected by RT-qPCR. **(E-H)** The positive expressions of LGMN, FN1, SRGN and CXCL16 in the IAs tissues and control tissues were detected by immunohistochemical staining. *p < 0.05, **p < 0.01, ***p < 0.001.

RT-qPCR was employed to detect the mRNA expression levels of relevant genes in 10 parietal tissues of IAs and 10 superficial temporal arteries. The expression levels of LGMN, FN1, SRGN and CXCL16 were found to be up-regulated in the parietal tissue of IAs (**Figures 11A–D**). We performed IHC to evaluate LGMN, FN1, SRGN and CXCL16 protein expression in IAs tissues and control tissues. The results showed that LGMN, FN1, SRGN and CXCL16 protein were significantly overexpressed in IAs tissues compared to neighboring normal tissues (**Figures 11E–H**).

## Discussion

A growing body of research suggests that IAs is considered a disease driven by chronic inflammation, with numerous studies examining the upregulation of inflammatory mediators, disruption of the elastic lining of the layer, and thinning of these mediators, including parietal cell death (27, 28), cellular and molecular inflammation plays a significant role in both aneurysm formation and rupture. Mo/MΦ play a significant role in the regulation of immune microenvironment and exert a long-lasting influence on the development and progression of IAs (29). Recent research findings have demonstrated that monocyte chemotactic protein-1 (MCP-1) serves as the principal chemotactic factor for macrophages, genetic deletion or inhibition of MCP-1, as well as pharmacological depletion of macrophages using clodronate liposomes, significantly impede the development and expansion of IAs (30, 31).

Many researchers have proposed an IAs correlation prediction model based on scRNA-seq; however, the comprehensive analysis of monocytes, particularly the investigation of monocyte biomarkers using scRNA-seq to analyze IAs immune infiltration and assess precision therapy, remains limited. In this study, we employed scRNA-seq and hdWGCNA to identify specific subsets associated with mononucleosis. The enrichment analysis of all genes in the resulting module was subsequently conducted. By conducting KEGG analysis, we have successfully identified the HIF-1 signaling pathway and NF−kappa B signaling pathway as pivotal pathways associated with Mo/MΦ heterogeneity. Gao et al (32). demonstrated that miR-4735 plays a pivotal role in the phenotypic regulation of vascular smooth muscle cells. Downregulation of miR-4735 expression in IAs tissues upregulates HIF-1 expression, thereby inducing autophagy activation. Enhanced autophagy facilitates cell proliferation and migration. This study has significant implications for elucidating the mechanism underlying IAs formation and suggests that targeting miR-4735 regulation, as well as HIF-1-mediated autophagy, could serve as a potential molecular therapeutic strategy to prevent IAs development. And the pathogenic role of the NF−kappa B signaling pathway in IAs involves various factors, which significantly contribute to the process of vascular remodeling by promoting it (33). The subsequent GO analysis revealed a strong association with neutrophil activation, neutrophil activation involved in immune response, neutrophil mediated immunity and neutrophil degranulation. The study conducted by Masaaki et al (34). demonstrated that neutrophil extracellular traps (NETs) can contribute to the rupture of intracranial aneurysms. Pharmacological intervention targeting peptidyl arginine deiminase 4 or the dissolution of pre-existing NETs could potentially mitigate this effect.

Next, by utilizing six machine learning methods, we successfully identified four characteristic genes for disease diagnosis and immune microenvironment analysis, namely LGMN, FN1, SRGN, and CXCL16. The four genes were subsequently assessed using ROC curves and expression profiles, revealing significant up-regulation in the IAs samples of both the training set and the validation set. The LGMN (Legumain) enzyme belongs to the peptidase family C13 and functions as an asparagine-specific cysteine endopeptidase (35). Extensive research has consistently demonstrated high expression of LGMN in monocytes and macrophages (M1 and M2) (36, 37), with its expression and secretion increasing during the differentiation process from monocytes to macrophages (36, 38), providing evidence for the involvement of LGMN in biological immune processes. The FN1 (fibronectin 1) is a crucial constituent of the extracellular matrix within the arterial wall, playing a pivotal role in pathological angiogenesis, embryonic blood vessel morphology, and maintenance of arterial wall integrity (39, 40). The high expression and localization of FN1 in IAs have been demonstrated, and based on the ROC results, FN1 exhibits a remarkable sensitivity and specificity in samples from IAs patients (39, 41, 42). This suggests that FN1 may play a direct role in the initiation and progression of IAs, thereby providing a crucial foundation for guiding targeted therapy strategies. The intracellular proteoglycan SRGN (serglycin) is synthesized by inflammatory and stromal cells. The production of serglycin -/- mice demonstrated the extensive effects of serglycin on the functional properties of immune cells. The serglycin molecule contains chondroitin sulfate chains, which are primarily sulfated at the C4 position of N-acetyl-galactosamine. Interestingly, the sulfation pattern of chondroitin sulfate in serglycin is regulated during monocyte differentiation into macrophages. CXCL16 is a chemokine composed of 254 amino acids that is widely expressed in immune cells such as monocytes, macrophages and B cells (43, 44). CXCL16 is crucial for immune cell adhesion to the endothelium and DC during inflammation. Numerous studies have demonstrated that CXCL16 plays a pivotal role in regulating immune response and mediating inflammation (45). Moreover, it plays a pivotal role in various autoimmune disorders as well as the regular functioning of the immune system (46). Additionally, CXCL16 significantly contributes to the progression of atherosclerosis (47), as evidenced by the observation that mice deficient in both CXCL16 and LDLR (CXCL16−/− low density and low intensity −/−) exhibit exacerbated atherosclerotic lesions primarily due to impaired cholesterol efflux caused by dysfunctional CXCL16 receptor activity (47, 48). A considerable body of clinical research has demonstrated that atherosclerosis constitutes one of the risk factors for IAs, and the presence of atherosclerotic plaque within the aneurysm wall may contribute to its degeneration and subsequent rupture (49, 50).

Finally, ROC curves and expression curves showed that LGMN, FN1, SRGN and CXCL16 were significantly up-regulated in both the training and validation sets. Subsequently, we further analyzed the difference between unruptured and ruptured aneurysms, and the results showed that ruptured IAs samples had higher levels of hub gene expression. This suggests that key genes may be involved in the formation and breakdown of IAs. We employed MCP-counter to conduct an analysis on the correlation between 10 distinct immune cell types and stromal cells as well as hub genes. Encouragingly, our findings aligned with our initial expectations, revealing a strong positive association between monocytes and both CXCL16 and LGMN. The subsequent analysis revealed that, in comparison to normal samples, nearly all of the 28 immune-related cells exhibited a significant upregulation in IAs samples, thereby further substantiating the notion that inflammation plays a pivotal role as an important pathogenic factor in IAs.

Although this study provides novel insights into targeted therapies for IAs, there are inherent limitations that need to be acknowledged. Firstly, In external validation, the accuracy was 70% in the training group and 100% in the validation group, which may be due to the small sample size in the dataset, so future models need to be further validated in a larger aneurysm cohort. Additionally, conducting more animal experiments is imperative to comprehensively understand the biological functionality of key genes. Then, this study only verified the expression of key genes without further analyzing this subpopulation and demonstrating its relevance to monocytes. Lastly, we only investigated the expression of key genes, but the relationship with monocytes has not been further confirmed; therefore, additional research is required to explore the protein expression levels of prognostic genes.

In conclusion, our study successfully identified disease-specific subgroups of Mo/MΦ and provided valuable insights into the crucial role of monocyte-associated genes in immune infiltration in IAs through the establishment of promising machine learning and deep learning models.

## Data availability statement

The datasets presented in this study can be found in online repositories. The names of the repository/repositories and accession number(s) can be found in the article/**Supplementary Material**.

## Ethics statement

The studies involving humans were approved by The Ethics Committee of the Affiliated Hospital of Qingdao University. The studies were conducted in accordance with the local legislation and institutional requirements. The participants provided their written informed consent to participate in this study.

## Author contributions

YX: Writing – review & editing, Writing – original draft, Formal analysis, Data curation, Conceptualization. PG: Formal analysis, Data curation, Writing – review & editing. GW: Conceptualization, Writing – review & editing. XS: Validation, Formal analysis, Writing – review & editing. CW: Resources, Writing – review & editing, Software. HL: Supervision, Software, Writing – review & editing. ZC: Visualization, Methodology, Writing – review & editing. PZ: Validation, Supervision, Writing – review & editing. YF: Methodology, Funding acquisition, Writing – review & editing.

## Supplementary material

Supplementary Figure 1 Single cell analysis of cell proportion changes. **(A)** Expression levels of marker genes. **(B)** Bar plot indicates the cell proportion of all ten cell types in IAs and shams.

Supplementary Figure 2 Construction of The gene-gene interaction network and correlation analysis of hub genes. **(A)** The GeneMANIA database was utilized for the analysis of the gene-gene interaction network involving hub genes. **(B)** Correlation analysis of hub genes.

Supplementary Figure 3 GSEA identifies signaling pathways involved in the diagnostic marker genes. **(A)** GSEA analysis of LGMN gene. **(B)** GSEA analysis of SRGN gene. **(C)** GSEA analysis of FN1 gene. **(D)** GSEA analysis of CXCL16 gene.
