# Supplementary figures and images for: Integrated analysis of single-cell sequencing and machine learning identifies a signature based on monocyte/macrophage hub genes to analyze the intracranial aneurysm associated immune microenvironment
Source: Front Immunol. 2024 Jun 24;15:1397475. doi: 10.3389/fimmu.2024.1397475 (PMC11228246; doi:10.3389/fimmu.2024.1397475)

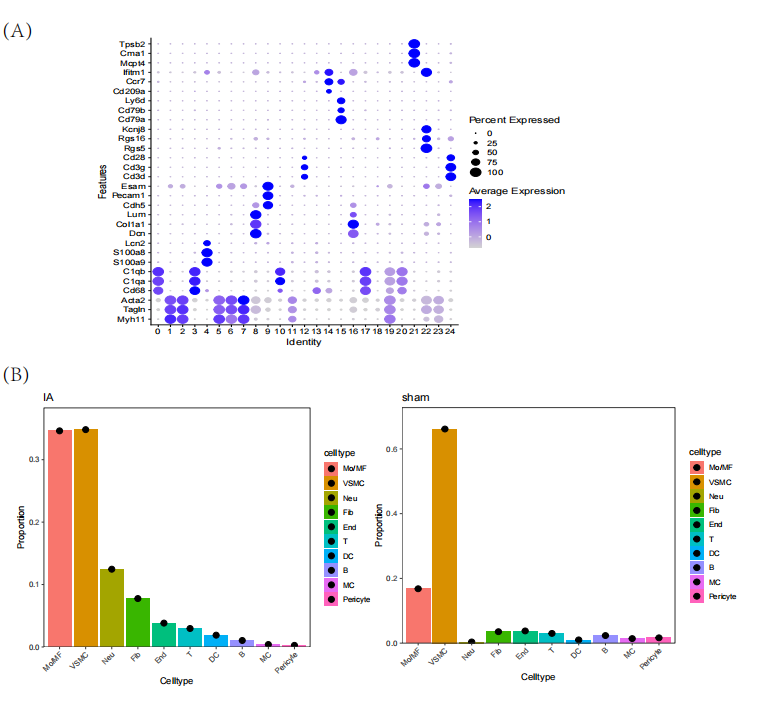

Supplement: Supplementary Figure 1 — Single cell analysis of cell proportion changes. (A) Expression levels of marker genes. (B) Bar plot indicates the cell proportion of all ten cell types in IAs and shams. [file Image_1.png]

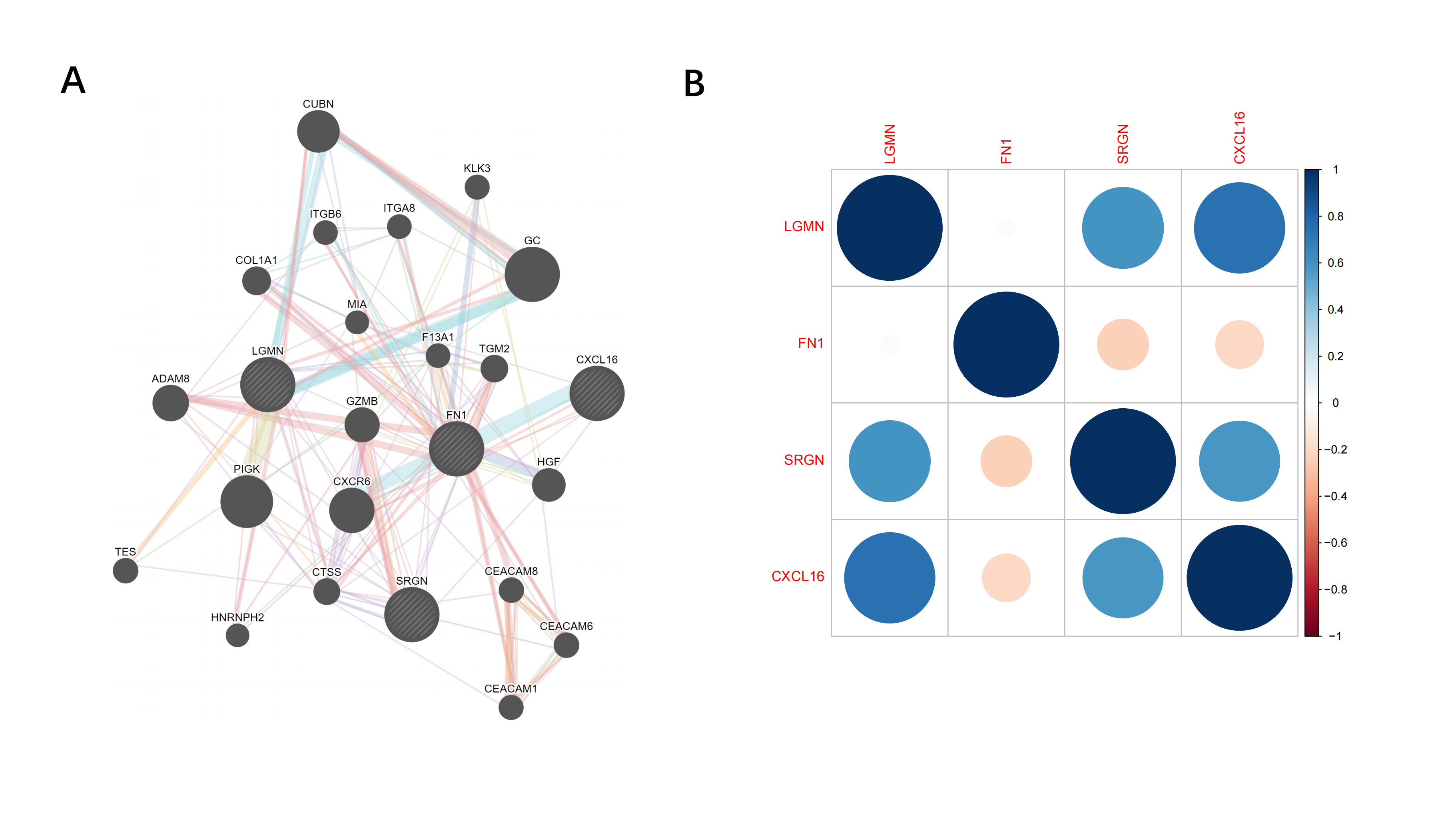

Supplement: Supplementary Figure 2 — Construction of The gene-gene interaction network and correlation analysis of hub genes. (A) The GeneMANIA database was utilized for the analysis of the gene-gene interaction network involving hub genes. (B) Correlation analysis of hub genes. [file Image_2.tif]

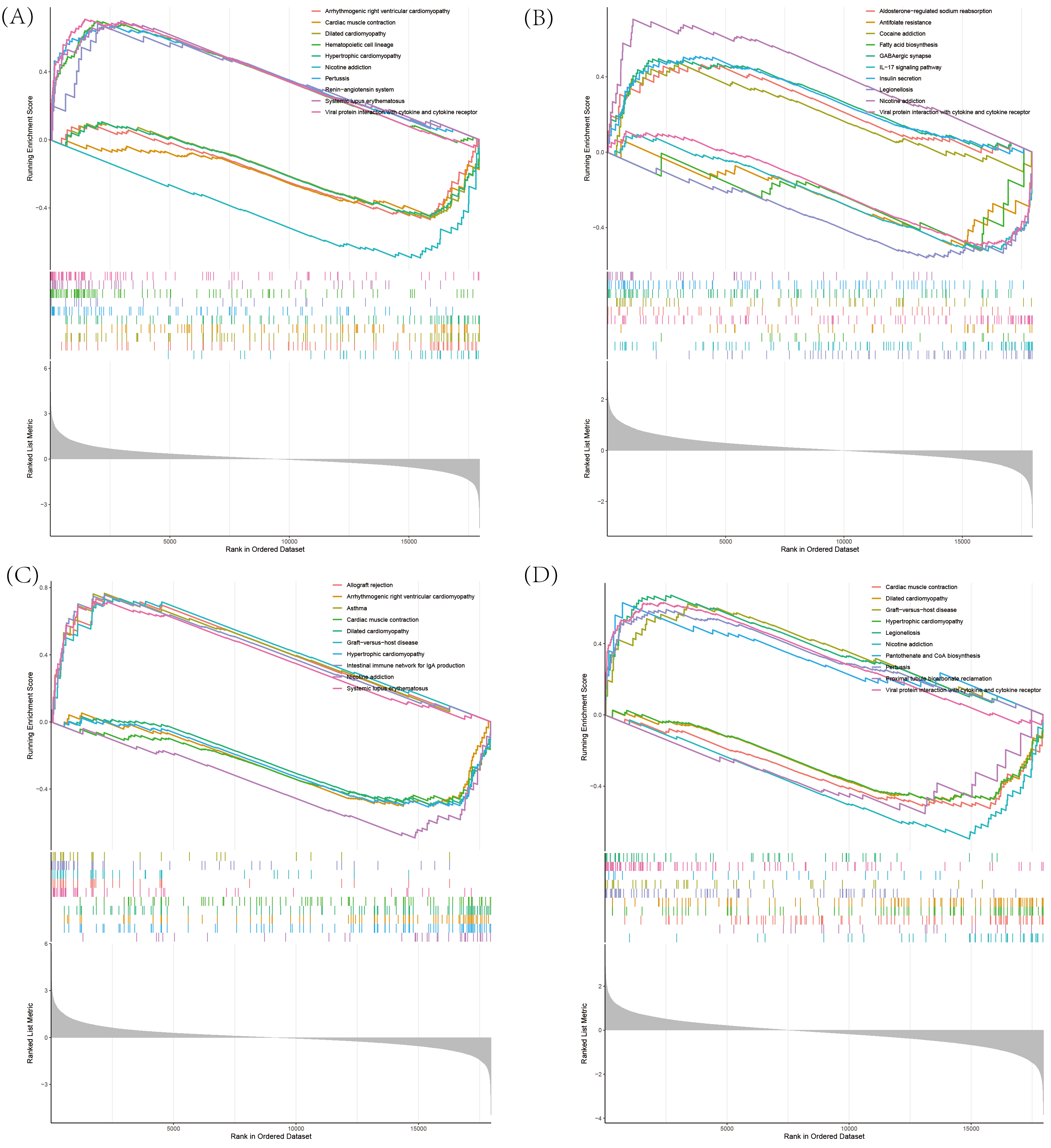

Supplement: Supplementary Figure 3 — GSEA identifies signaling pathways involved in the diagnostic marker genes. (A) GSEA analysis of LGMN gene. (B) GSEA analysis of SRGN gene. (C) GSEA analysis of FN1 gene. (D) GSEA analysis of CXCL16 gene. [file Image_3.png]
