# Supplementary material for: Integrated analysis of single-cell sequencing and machine learning identifies a signature based on monocyte/macrophage hub genes to analyze the intracranial aneurysm associated immune microenvironment
Source: Front Immunol. 2024 Jun 24;15:1397475. doi: 10.3389/fimmu.2024.1397475 (PMC11228246; doi:10.3389/fimmu.2024.1397475)
Supplement: Supplementary file 7 [file Table_4.docx]

| Patient | Gender | Age | Hypertension |
| --- | --- | --- | --- |
| IAs1 | Female | 71 | √ |
| IAs2 | Female | 67 | √ |
| IAs3 | Female | 65 | √ |
| IAs4 | Male | 62 | √ |
| IAs5 | Male | 73 | √ |
| Control1 | Female | 70 | √ |
| Control2 | Female | 66 | √ |
| Control3 | Female | 70 | √ |
| Control4 | Male | 69 | √ |
| Control5 | Male | 62 | √ |

We matched the experimental group and the control group according to sex, age and hypertension, in which the experimental group and the control group had three women and two men, and all were elderly patients (over 60 years old) with hypertension.
